# The Impact of Weighted Blanket Use on Adults with Sensory Sensitivity and Insomnia

**DOI:** 10.1155/2023/3109388

**Published:** 2023-12-20

**Authors:** Rhonda Davis-Cheshire, Savannah Bennington, Allison Hartsek, Teresa Kelly, Janeene Marinelli, Amanda Perez

**Affiliations:** Kettering College, 3737 Southern Blvd, Kettering, OH 45429, USA

## Abstract

**Purpose:**

This study's purpose was to determine the impact of weighted blanket use on moderate to severe insomnia in adults with sensory sensitivity greater than the average population.

**Methods:**

For this study, a four-week, single-case, multiple-participant ABA study design was used. Through convenience sampling, four participants scoring 15 or greater on the Insomnia Severity Index (ISI), which categorizes them as having moderate to severe insomnia, and much more than most people in sensory sensitivity on the Adolescent/Adult Sensory Profile were recruited. First, seven-day baseline sleep data was gathered, followed by two weeks of weighted blanket use, concluding with a seven-day withdrawal phase. Additional outcome measures included: Tuck and Snooze Survey, Consensus Sleep Diary Morning, and Additional Sleep Diary Questions. Data analysis included visual analysis, mean comparisons, Tau-*U* calculations, and pre- to post-ISI category comparisons.

**Results:**

All participants' ISI scores were categorized as one level less severe postintervention. All participants demonstrated increased sleep quality, and three participants showed an increase in sleep duration based on individual mean comparisons between baseline and intervention phases.

**Conclusion:**

Weighted blankets appear beneficial in reducing insomnia severity in adults with much more than the average population sensory sensitivity. In addition, those with self-reported anxiety may have increased benefit from this intervention.

## 1. Introduction

Sleep difficulties impact many individuals worldwide, with between 50 and 70 million adults experiencing sleeping difficulties in the United States alone [1], approximately 30% of the adult population. Quality sleep has been found to be a strong indicator of both mental and physical health [2]. There are numerous impacts of sleep disorders on health such as anxiety, depression, impaired cognition, hypertension, cardiovascular disease, weight gain, and type II diabetes [3]. In addition, the cost of sleep disorders is significant with a recent Australian study reporting that an estimated $35.4 billion was spent in Australia addressing this issue in 2019-2020 when combining expenses for health care, lost productivity, etc. [4]. Health risks associated with poor sleep continue into adulthood, and sleep disorders worsen over time, which can be associated with lifestyle choices [5].

## 2. Literature Review

### 2.1. Factors That Contribute to Adult Sleep Difficulties

Several researchers have explored the causes of adult sleep issues. Fatima et al. [6] identified multiple factors that contribute to poor sleep quality in adults including low socioeconomic status, an unhealthy lifestyle, poor health, ethnicity, shift work, depressive symptoms, and obesity. Lund et al. [7] found that, for college students, emotional and academic stress had the largest negative impact on sleep while the use of prescription, over-the-counter, and recreational drugs, as well as alcohol consumption, also impacted sleep. Gender impacts sleep, as Fatima et al. [6] reported that adult females have poorer sleep quality than their male counterparts and Tsai and Li [8] found that female college students tend to have poorer sleep quality compared to their male peers. In addition, Fatima et al. [6] illuminated how these factors associated with poor sleep are often interrelated, experienced simultaneously, and can have a compounding impact on adult sleep quality.

### 2.2. Interventions to Improve Sleep Quality

Besides medications for insomnia and specific medical interventions for apnea, a variety of interventions to improve sleep quality have been studied including sleep hygiene education [9, 10], cognitive behavioral therapy [11], mindfulness meditation via the app Calm [12], sleep education via text messaging [13], and environmental modifications such as the use of a sunrise alarm clock [14] or music [15, 16] with varying degrees of success.

Occupational therapists in the United States consider sleep and rest to be an essential occupation with the *Occupational Therapy Practice Framework: Domain and Process 4^th^ ed*. [17] describing quality sleep as a support for participation in other occupations. Recent occupational therapy studies have assessed the impact of sleep interventions including the impact of napping [18], sleep preparation utilizing sleep hygiene, Dreampad Pillow® (a sleep aid that plays calming music), and iRest® meditation [19] and the impact of utilizing a Restoring Effective Sleep Tranquility program (compilation of cognitive behavioral therapy approaches) [20].

### 2.3. The Impact of Sensory Processing Differences on Sleep Quality

One factor that appears to contribute to poor sleep quality is the presence of certain sensory differences due to variations in sensory processing [21]. Children with certain sensory differences are more likely to experience poor sleep quality than peers without these differences [21, 22]. Shochat et al. [21] found that children with sensory differences, especially tactile sensitivity, were more likely to have sleep difficulties. Rajaei et al. [22] found significant differences in sensory patterns in children who had sleep disorders compared to those who did not. Tzischinsky et al. [23] found the strongest correlation between a specific sensory difference, touch hypersensitivity, and increased sleep disturbances in children with autism spectrum disorder (ASD). Children in this study without ASD also demonstrated a correlation, though weaker, with touch hypersensitivity and sleep disturbances. Mazurek and Petroski [24] found a significant relationship between sensory over-reactivity and sleep difficulties.

In the adult population, Engel-Yeger and Shochat [25] found that poor sleep quality in healthy adults was significantly correlated with sensory avoidance and sensory sensitivity based on participant reports on the Adolescent/Adult Sensory Profile (AASP) and Pittsburgh Sleep Quality Index (PSQI). Tactile sensitivity was most predictive of sleep difficulties for participants in results on the sensory sensitivity section of the AASP. The researchers concluded that these findings suggested that poor sleep quality may correlate with low sensory thresholds. Sharfi and Rosenblum [26] found that adults with learning disabilities were significantly more likely than typical peers to have sensory processing differences and poor sleep quality, with sensory sensitivity and low registration being significant indicators of sleep quality based on participant report on the AASP and Mini Sleep Questionnaire. Additionally, Hohn et al. [27] illuminated a link between visual sensory hyper-reactivity and increased insomnia severity among adults with ASD based on results from the Sensory Perception Quotient and the Insomnia Severity Index (ISI).

### 2.4. Weighted Blanket Use

Weighted blankets provide proprioceptive input also described as deep pressure touch [28–30]. Deep pressure touch (DPT) is created with sensations such as firm touching, holding, and swaddling and has been noted to have a relaxing and calming effect on the nervous system [31]. When utilizing weighted vests to provide DPT to impact child behaviors, attention, etc., often the vest weight is recommended to be approximately 10% of the individual's body weight [32] though evidence to support the effectiveness of weighted vests is limited [33]. Based on these vest weight recommendations, some implementing weighted blanket researches have used this 10% of body weight target [34–37]. Over the past decade, weighted blankets have gained more widespread attention and consumer availability and with this an increase in studies assessing their impact. For example, several researchers have examined the effectiveness of using a weighted blanket on adults during the daytime to reduce anxiety which resulted in a significant decrease in anxiety [28–30]. In addition, occupational therapists as well as other professionals have begun to assess the impact of weighted blanket use to specifically address nighttime sleep difficulties [34–36, 38–41].

Ackerley et al. [38] conducted a four-week study with 31 adults who had significant sleep disturbance incorporating a chain-weighted blanket and found, based on actigraphy and polysomnography results, a statistically significant increase in sleep duration and decrease in activity while in bed when participants used the weighted blankets and, per the Karolinska Sleepiness Scale, an improvement in sleep quality. Ekholm et al. [39] completed an RCT examining the impact of weighted blanket use on the nighttime sleep of 120 adults. Besides insomnia, inclusion criteria for this study also required participants have one of the following psychiatric diagnoses: attention deficit hyperactivity disorder, bipolar disorder, generalized anxiety disorder, or major depression. Pre- to postresults from the ISI revealed a significant decrease in insomnia symptoms following one week of blanket use as well as a group mean ISI category changes from severe to subthreshold insomnia.

### 2.5. Weighted Blanket Use for Those with Sensory Differences

Occupational therapists have historically been leaders in identifying and treating individuals with sensory processing differences [42]. Occupational therapists use a variety of sensory modulation strategies in practice to support occupational participation for those with sensory differences, including incorporating DPT, such as utilizing weighted blankets, weighted vests [43], or weighted wraps [44]. The use of weighted blankets as an intervention to enhance nighttime sleep for children with ASD, a population that frequently presents with sensory processing differences [45], has begun to be explored [34–36, 41].

A succession of single-subject ABA design studies has been completed examining weighted blanket use to enhance the sleep of children diagnosed with ASD who also exhibited sensory over-responsivity in the tactile and/or auditory areas, as determined by the Sensory Processing Measure or Sensory Processing Measure-Preschool version [34–36]. Gee et al. [34] included two participants, and based on results of a parent online survey, use of the weighted blanket did minimally impact participant sleep quality with increased total sleep amount and decreased time to fall asleep. The Gee et al. [36] and Gee et al. [35] studies both included two participants and utilized the Sense Sleep App and the Daily Caregiver Survey as outcome measures. The Gee et al. [36] results indicated that one participant experienced an increase in total sleep time, while the other participant exhibited a decrease in the time needed to fall asleep. The Gee et al. [35] results were mixed: one participant showed an increase in overall time asleep and fewer awakenings, and the other participant experienced an improvement in morning mood.

Gringas et al. [41] conducted a RCT examining the impact on total sleep time when using weighted blankets on 63 children diagnosed with ASD. Unlike Gee et al. [34], Gee et al. [35], and Gee et al. [36], this study did not include a sensory assessment component as part of their participant inclusion criteria. Based on actigraphy and participant sleep diaries, there was no significant difference in total sleep time when comparing use of a traditional blanket to use of the weighted blanket, although, based on the questionnaire results, most parents (51%) of children in this study indicated a belief that their child's sleep was better when using the weighted blanket.

In the adult population with sensory differences, Green et al. [46] completed a qualitative study regarding weighted blanket use that included 16 participants with ASD who self-reported sensory differences. Participants in this study indicated that they used weighted blankets for nighttime sleep as well as during periods of anxiety. Participants reported greater sleep time with fewer sleep interruptions when using weighted blankets.

### 2.6. Purpose

Sleep difficulties are experienced by many people over the lifespan and a variety of interventions to mitigate issues with sleep quality and quantity have been explored. Research has indicated a correlation between individuals with sensory differences, particularly tactile over-reactivity, and sleep problems [23, 25]. Some initial studies attempting to address the sleep problems of individuals with sensory differences using weighted blankets have been completed targeting children with ASD and sensory over-reactivity, tactile and/or auditory [34–36], and adults with self-reported sensory differences [46]. Eron et al. [40] indicated in their systematic review of weighted blanket use how there are no clear evidenced-based guidelines for when to use this sensory modulation approach with clients. For occupational therapists, it is essential that assessment and documentation of a pattern of sensory differences are completed prior to providing sensory interventions [47], such as a weighted blanket, so that interventions are appropriately matched to the client. The purpose of this study was to explore the impact of the use of weighted blankets as an intervention for adults with moderate to severe insomnia and sensory sensitivity during nighttime sleep to determine which individuals might benefit from this sensory modulation intervention.

## 3. Methods

### 3.1. Research Design

We used a single-case, multiple-participant ABA design for this study. This design has been used previously to explore the impact of weighted blanket use on the sleep of children with an ASD diagnosis [34–36]. This approach is appropriate for analysis of an individual as well as a small participant group [48] and is suitable for early-stage clinical intervention research. This method was chosen to determine the effectiveness of a weighted blanket intervention while evaluating a variety of other variables impacting sleep quality. This study underwent a full review and received approval from the Kettering Health Institutional Review Board, and all participants gave informed consent.

### 3.2. Participants

#### 3.2.1. Recruitment

The researchers sought a convenience sample of subjects via the researchers' personal social media accounts, word of mouth, and through direct emails to acquaintances, friends, and family. Social media platforms included Facebook, Instagram, Snapchat, and Twitter. Word of mouth included in-person conversations and virtual communication via telephone calls, texting, and video calls. Recruitment materials included a flyer and standardized email both containing study details, general description of sleep difficulties and sensory sensitivities researchers were looking for in participants, and inclusion and exclusion criteria. Researchers distributed the flyer on social media platforms and encouraged sharing of the flyer to others for further recruitment. Researchers used their own greetings and closures for recruitment outreach including hashtags in social media posts.

The study was designed to allow up to five participants. Potential participants who contacted the researchers to be considered for this study were first provided the ISI via email, and if these potential participants scored 15 or greater on the ISI (categorizing them as having moderate to severe insomnia), they were next provided the AASP. The researchers sent out 38 ISI assessments, and 26 were received for scoring. Based on ISI results, the researchers sent out 24 AASP assessments of which 8 participants returned and scored in the sensory sensitivity section as *much more than most people*, qualifying them for this study. These participants were sent study consent forms with the first five who returned completed forms accepted into the study. Recruitment was a five-week process.

#### 3.2.2. Inclusion Criteria

Participants in this study needed to be at least 18 years of age, be fluent in reading and writing English, and living in the contiguous United States. In addition, participants needed to score 15 or greater on the ISI (indicating moderate to severe insomnia) as well as score in the much more than the average population on the sensory sensitivity category of the AASP.

#### 3.2.3. Exclusion Criteria

Participants were excluded if they self-identified as having been diagnosed with sleep apnea, untreated metabolic disorders, and untreated high blood pressure, were pregnant, or had open wounds. Additionally, people did not qualify if they self-identified as currently using a weighted blanket were physically unable to move or remove the weighted blanket independently, reported abusing alcohol or illicit drugs, or were currently participating in another sleep study.

### 3.3. Instruments and Outcome Measures

The ISI [49] is a self-report questionnaire containing seven items to assess the impact, nature, and severity of insomnia. This questionnaire is representative of the past two weeks of sleep for the individual. The ISI contains a five-point Likert scale (0 = no problem; 4 = severe problem) used to rate the severity of different sleep problems. Total scores range from zero to 28. Total scores are interpreted as absence of insomnia, subthreshold insomnia, moderate insomnia, or severe insomnia. Morin et al. [49] reported high internal consistency coefficients from clinical and community samples and, when considering criterion validity, for the ISI cutoff score > 8 (subthreshold insomnia), the clinical sample yielded a 99.4% sensitivity and 91.8% specificity, and the community sample yielded an 95.8% sensitivity and 78.3% specificity. Congruent validity was found in comparison to PSQI total scores, showing r958 = 0.80, *p* < 0.05, suggesting good congruent validity [49]. This tool was used as part of the initial screening to determine participant inclusion as well as a postintervention measure.

The AASP [50] is a 60-item standardized self-questionnaire that collects information on sensory processing patterns of individuals in the areas of auditory processing, taste/smell processing, movement processing, visual processing, touch processing, and associated activity levels. Subjects indicate how frequently they respond to various sensory events, almost never to almost always. Questions are scored on four subscales: low registration, sensation seeking, sensory sensitivity, and sensation avoiding. Individual total scores place them into one of five categories of sensory processing patterns: much less than most people, less than most people, similar to most people, more than most people, and much more than most people. The AASP can be administered to individuals aged 11 years and older. The reliability of the AASP is demonstrated by the coefficient alpha of 0.639 to 0.775, with one signifying perfect reliability [50]. The AASP has strong construct validity [50]. This tool was used at screening to determine participant inclusion.

The researchers created the Tuck and Snooze Survey (TSS), an 18-item study-specific survey, to gather more extensive background information from participants related to sleep (see Table 1). This survey gleans additional participant data related to demographics, incidence and type of physical exercise, anxiety, sleep environment, and sleep routines. The survey has multiple choice questions (*n* = 11), check-all-that-apply questions (*n* = 4), and short-answer demographic questions (*n* = 3). Participants completed this tool prior to the start of the sleep intervention.

The Consensus Sleep Diary Morning (CSDM) [51] is an outcome measure consisting of 15 questions about nighttime sleep experiences for the participant to fill out daily, after waking the following morning. The CSDM contains specific directions and examples for each question, along with definitions of otherwise subjective terminology, such as the words “bed” and “day.” The CSDM includes questions related to sleep quantity, perceived quality, number and duration of awakenings during the night, sleep onset latency, quantity and duration of naps, consumption of caffeine and alcohol, information about any sleep medications taken, and a space for participant comments. Sleep quality is measured on a five-point Likert scale with one being very poor and five being very good. Reliability and validity for the CSDM have not been formally established and have been difficult to establish because sleep is never the same over multiple nights [51]. In a study by Dietch and Taylor [52], sleep midpoint, total sleep time, and sleep efficacy were measured by the Consensus Sleep Diary (CSD) as well as actigraphy and EEG. The data from the CSD was more aligned with the actigraphy than the EEG, showing good validity. However, this study was not a formal evaluation of the validity of the CSD [52]. This tool was used daily throughout the study to provide data on participant sleep.

The Additional Sleep Diary Questions (ASDQ), a six-item survey, was created by the researchers for this study to supplement the CSDM (see Table 2). This measure was created to extract any other variables impacting sleep that the researchers thought might be relevant that were not addressed in the CSDM. The ASDQ included questions regarding medications with side effects related to sleep, adherence to using the weighted blanket, any environmental sleep disruptions, and exercise out of a normal routine. This survey included three yes/no and three fill-in-the-blank questions. The ASDQ was completed daily throughout the study.

### 3.4. Procedures

The consenting participants were mailed an Essentials by Tranquility, 48 by 72 inch, 12-pound weighted blanket. Because there is almost no evidence to support using weighted blankets that are 10% of body weight since they are based on weighted vest use, and because weighted vests only cover the torso and are typically worn when upright, whereas a blanket would cover the entire body while supine, the researchers opted to utilize a lighter weight blanket that is easily available to consumers for this study. Since evidence is needed to determine optimal blanket weight, the researchers determined to assess if the 10% recommendation being adopted for weighted blanket use might be unnecessarily heavy to impact insomnia. The researchers desired to evaluate the least invasive or lowest dose option.

Participants were also mailed a packet of study-specific instructions and study outcome measures including the TSS, CSDM, ASDQ, and the ISI as well as a return addressed stamped envelope. Participants initiated the study within four weeks of qualifying and consenting. Each start date was individualized based on receipt of study items and participant preference within study parameters. The TSS was completed by participants prior to the start of the 4-week intervention. Week one of the study established a baseline for participants who slept as usual without the weighted blanket and completed the CSDM and ASDQ upon waking every day. In weeks two and three of the study, participants used the weighted blanket during nighttime sleep only then completed the CSDM and ASDQ upon waking every day. At the end of the intervention period, on study day 21, participants completed the ISI. Week four of the study was a withdrawal period with participants returning to sleep as usual without the weighted blanket, still completing the CSDM and ASDQ daily upon waking. Upon completion of the study, participants returned the TSS, CSDM, ASDQ, and ISI to the researchers via mail.

### 3.5. Data Analysis

AASP scores as well as pre- and post-ISI scores were calculated and categorized. Visual analysis of level changes comparing daily outcome measures at three phases, baseline, intervention, and withdrawal as well as direction of trend-line slopes was completed using charts created through Microsoft Excel. Individual mean comparisons for sleep quality and quantity from the CSDM were completed for all phases. Tau-*U* calculations were completed for sleep quality and quantity based on results from the CSDM comparing baseline to intervention phases utilizing the free online single-case research calculator [53] to determine the effect of the intervention. Baseline Tau-*U* calculation corrections were completed when a baseline Tau was > 0.40 as recommended by Parker et al. [54]. Per Vannest and Ninci [55], the Tau-*U* values for this study comparing baseline to intervention were interpreted as a large to very large effect if they were >0.80, they were interpreted as a large effect if they were from 0.60 to 0.80, they were considered a moderate effect if they were from 0.20 to 0.60, and they were considered a small effect if they were at 0.20. A *p* value < 0.05 was considered significant.

## 4. Results

### 4.1. Demographics

Five participants qualified and consented to participate in this study and were provided weighted blankets and study outcome measures. One participant ceased contact with the researchers after receiving materials and did not return any completed outcome measures, and therefore, the study consisted of the remaining four participants who were all female ranging in age from 21 to 46 years. Two participants resided in the state of Ohio, one resided in Pennsylvania, and another in New York.

### 4.2. Tuck and Snooze Survey

Specific demographic data was gathered via the Tuck and Snooze Survey for all four participants (see Table 1). Participant 1 (P1) was a 21-year-old female working as a waitress and indicated she occasionally exercised. Based on her reported height and weight, her body mass index (BMI) score placed her in the healthy range [56]. The weighted blanket was 9.6% of P1's body weight. She indicated she slept with mild bedroom lighting at night. P1 disclosed she worries frequently and has difficulty controlling her worries. Additionally, she self-identified as having anxiety, being diagnosed or treated for anxiety in the last 12 months and having taken prescription medication to manage her anxiety, and reported she thought anxiety might be contributing to her sleep issues.

Participant 2 (P2) was a 24-year-old female travel nurse who participated in exercise one to three times a week. Based on her reported height and weight, her BMI placed her in the obese range [56]. The weighted blanket was 6% of P2's body weight. P2 reported her sleeping environment was completely dark, and she used a sound machine to help her sleep. P2 indicated that she worries frequently and has difficulty controlling her worries. P2 self-identified as having anxiety, being diagnosed or treated for anxiety in the last 12 months and having taken prescription medication to manage her anxiety, and reported she thought anxiety might be contributing to her sleep issues.

Participant 3 (P3) was a 26-year-old female physician assistant graduate student, who reported exercising four to five times a week. Based on her reported height and weight, her BMI placed her in the healthy range [56]. The weighted blanket was 8.88% of P3's body weight. P3 indicated her bedroom environment was completely dark with a fan producing noise nightly, in addition to the occasional ambulances or helicopters that would produce noise outside of her room. P3 indicated she sometimes worries frequently and sometimes has difficulty controlling her worries. P3 self-identified as having anxiety and reported she thought anxiety might be contributing to her sleep issues; however, she did not report being diagnosed with or treated for anxiety within the past 12 months.

Participant 4 (P4) was a 46-year-old female working as an administrative assistant who reported exercising four to five days a week. Based on her reported height and weight, her BMI placed her in the overweight range [56]. The weighted blanket was 7.5% of P4's body weight. She slept with another person in the bed and reported occasionally having trouble sleeping due to the noise of the cosleeper. P4 also reported she was kept awake occasionally by the sounds of her neighbors. P4 indicated she sometimes worries frequently and sometimes has difficulty controlling her worries. P4 reported she thought anxiety might be contributing to her sleep issues; however, she did not self-identify as having anxiety or report being diagnosed with or treated for anxiety within the last 12 months.

### 4.3. AASP

The AASP sensory sensitivity scores for all four participants, as part of qualifying for this study, fell within the much more than most people category and were as follows: P1 was 56, P2 was 54, P3 was 51, and P4 was 57. Results also indicated two participants fell within the much more than most people category for low registration as follows: P1 was 50 and P2 was 48; and the remaining participants fell into the more than most people as follows: P3 was 38 and P4 was 36. Three of the participants' results for sensation avoiding placed them in the much more than most people category as follows: P2 was 57, P3 was 57, and P4 was 62, with the remaining participant falling within the more than most people category for sensation avoiding: P1 was 44. For the category sensation seeking, two participants fell within the category similar to most people: P2 was 47 and P4 was 44, while P1 fell into the less than most people category with 41 and P3 fell into the much less than most people category with 27.

### 4.4. ISI

**Figure 1:**
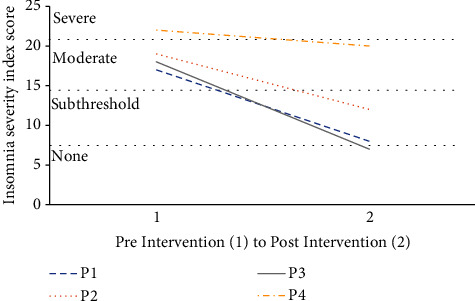
ISI change following weighted blanket intervention.

Participant preintervention baseline ISI scores categorized three participants (P1, P2, and P3) with moderate insomnia, scores ranging from 15 to 21, and one participant (P4) with severe insomnia, scores ranging from 22 to 28. Postintervention ISI scores decreased from baseline which resulted in a change in descriptive categories for all participants (see Figure 1). P1 had a baseline ISI score of 17, indicating moderate insomnia, and a postintervention ISI score of 8, indicating subthreshold insomnia. P2 had a baseline ISI score of 19, indicating moderate insomnia, and a postintervention ISI score of 12, indicating subthreshold insomnia. P3 had a baseline ISI score of 18, indicating moderate insomnia, and a postintervention ISI score of 7, indicating an absence of insomnia. P4 had a baseline ISI score of 22, indicating severe insomnia, and a postintervention ISI score of 20, indicating moderate insomnia.

### 4.5. CSDM

Participants fully completed the CSDM daily as requested except for P2 who did not provide any CDSM data for day 13 of their study which occurred during the intervention period. The following analysis is based on the CDSM data provided.

#### 4.5.1. Sleep Medications

One participant (P3) reported taking melatonin (5 mg) on two nights during the withdrawal period. No other participants reported taking medications to aide their sleep during this study.

#### 4.5.2. Sleep Quantity Visual Analysis and Mean Comparisons

**Figure 2:**
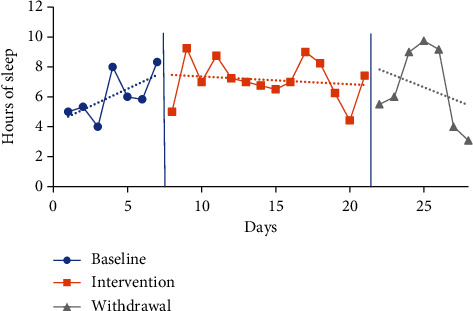
Quantity of sleep for P1.

**Figure 3:**
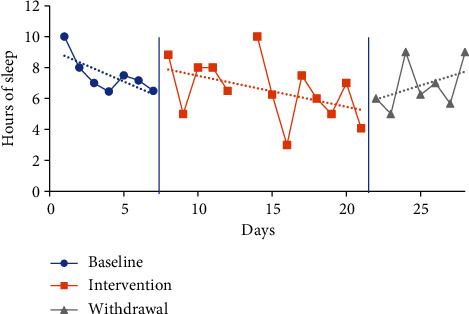
Quantity of sleep for P2.

**Figure 4:**
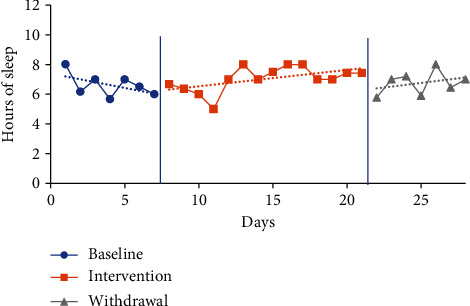
Quantity of sleep for P3.

**Figure 5:**
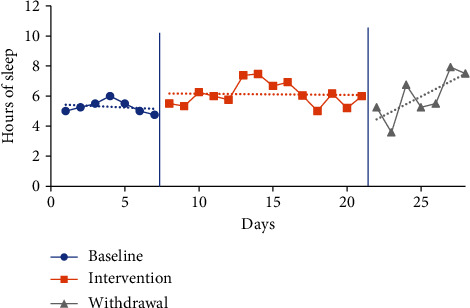
Quantity of sleep for P4.

The trendline for hours of sleep during the baseline period increased for P1 and decreased for P2 and P3 and slightly decreased for P4. The trendline for hours of sleep during the intervention phase increased for P3, decreased for P2, and slightly decreased for P1 and P4. The trendline for sleep duration during the withdrawal phase decreased for P1 and increased for P2, P3, and P4. No level change for sleep quantity was noted between phases for any participants. Individual participant results for sleep quantity are presented in Figures 2–5.

The mean sleep time for the baseline period was calculated based on a self-report from the CSDM (see Table 3) for all participants: P1 6.07 hours (4-8.33), P2 7.52 hours (6.45-10), P3 6.62 hours (5.67-8.02), and P4 5.29 hours (4.75-6). The intervention period mean sleep times were 7.13 hours (4.42-9.25) for P1, 6.55 hours (3-10) for P2, 7.03 hours (5-8) for P3, and 6.12 hours (5-7.47) for P4. The mean sleep time increased from baseline to intervention phase for P1 by 1.06 hours, P3 by 0.41 of an hour, and P4 by 0.83 of an hour. In comparison, the mean sleep time for P2 decreased from baseline to intervention by 0.97 hours.

The mean sleep time during the withdrawal phase decreased for three participants (P1, P3, and P4) and increased for one participant (P2) from the intervention phase. Withdrawal period mean sleep times were 6.64 hours (3.08-9.75) for P1, 6.85 hours (5-9) for P2, 6.76 hours (5.77-8) for P3, and 5.96 hours (3.58-7.92) for P4. P1 decreased by 0.49 of an hour, P3 by 0.27 of an hour, and P4 by 0.16 of an hour, and P2 increased by 0.3 of an hour.

#### 4.5.3. Sleep Quality Visual Analysis and Mean Comparisons

**Figure 6:**
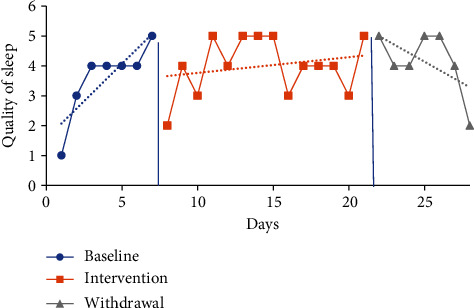
Quality of sleep for P1.

**Figure 7:**
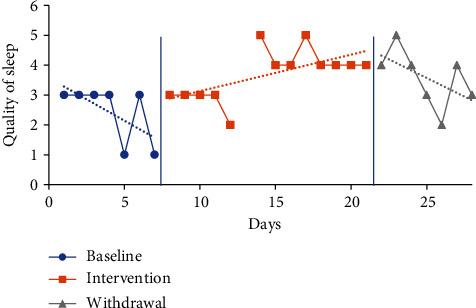
Quality of sleep for P2.

**Figure 8:**
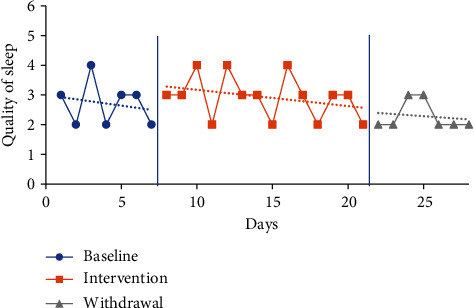
Quality of sleep for P3.

**Figure 9:**
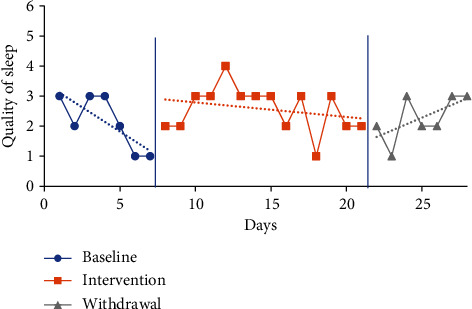
Quality of sleep for P4.

The trendline for quality of sleep during the baseline period increased sharply for P1 (indicating improved quality) and decreased for all other participants. The trendline for quality of sleep during the intervention phase increased for P1 and P2 (indicating improved quality) and declined for P3 and P4. The trendlines for the withdrawal phase declined for P1, P2, and P3 (indicating decreased quality) and increased for P4. No level change for sleep quality was noted between phases for any participants. Individual participant results for sleep quality are presented in Figures 6–9.

The mean sleep quality on the CDSM for all phases was calculated based on self-report via a five-point Likert scale with numerical report translating as follows: very poor (1), poor (2), fair (3), good (4), and very good (5). The following were the CDSM sleep quality reports for all participants (see Table 4) at baseline: P1 3.57 (1-5), P2 2.43 (1-3), P3 2.71 (2-4), and P4 2.14 (1-3). The mean sleep quality ratings during the intervention phase were 4 (2-5) for P1, 3.69 (2-5) for P2, 2.92 (2-4) for P3, and 2.57 (1-4) for P4. Mean sleep quality improved when comparing intervention to baseline period for all participants. P1 increased by 0.43 moving from the fair to good category, P2 increased by 1.26 moving from the poor to fair category, P3 increased by 0.21, and P4 increased by 0.43 with neither changing category from baseline.

The mean sleep quality ratings during the withdrawal phase were 4.14 (2-5) for P1, 3.57 (2-5) for P2, 2.29 (2-3) for P3, and 2.29 (1-3) for P4. During the withdrawal phase, mean sleep quality declined for P2, P3, and P4 and improved for P1. P1 increased by 0.14, while P2 decreased by 0.12, P3 decreased by 0.63, and P4 decreased by 0.28.

#### 4.5.4. Tau-*U* Analysis

Tau-*U* analysis of intervention effect was completed for sleep quantity and quality based on participant self-report from the CSDM comparing baseline to intervention phase for the individuals as well as an aggregated group. Baseline corrections were completed for P1 and P2 for sleep quantity and P1 and P4 for sleep quality. For sleep quantity (see Table 5), P1 Tau was 0.31 (*z* = 1.12, *p* = 0.26) indicating a moderate effect that was not statistically significant. For P2, the sleep quantity Tau was 0.19 (*z* = −0.67, *p* = 0.50) indicating no effect which was not statistically significant. For P3, the sleep quantity Tau was 0.32 (*z* = 1.16, *p* = 0.25) indicating a moderate effect which was not statistically significant. For P4, the sleep quantity Tau was 0.67 (*z* = 2.46, *p* = 0.01) indicating a large effect that was statistically significant. The aggregated Tau for sleep quantity of participants was 0.28 (*z* = 2.03, *p* = 0.04) indicating a moderate effect which was statistically significant.

For sleep quality (see Table 6), P1 Tau was 0.03 (*z* = 0.11, *p* = 0.91) indicating no effect which was not statistically significant. For P2, the sleep quality Tau was 0.67 (*z* = 2.42, *p* = 0.02) indicating a large effect that was statistically significant. For P3, the sleep quality Tau was 0.16 (*z* = 0.60, *p* = 0.55) indicating no effect which was not statistically significant. For P4, the sleep quality Tau was 0.38 (*z* = 1.38, *p* = 0.17) indicating a moderate effect which was not statistically significant. The aggregated Tau for sleep quality of all participants was 0.31 (z = 2.25, *p* = 0.02) indicating a moderate effect which was statistically significant.

### 4.6. ASDQ

Results from the ASDQ included information about additional medications as well as indicating anything that might disrupt nighttime sleep. P1 indicated taking medication (acetaminophen, valacyclovir) during the baseline phase on two separate days. P1 reported toe pain one day during the baseline phase, as well as her cats, and having a nightmare disrupting her sleep one day each during the intervention phase.

P2 reported taking Excedrin Migraine one day during the baseline phase. P2 reported various sleep disruptions throughout the baseline phase including family noise on three days, being too hot on two days, light on watch, alarm, and having side effects from taking a COVID-19 booster. During the intervention phase, P2 reported being too hot and too cold on 1 day and the noise of an alarm. During the withdrawal phase P2 reported being too hot on three days, having a runny/blowing nose on two days, and a light in the hall on one day as disrupting her sleep.

P3 reported taking fluticasone propionate nasal spray on one day during the baseline phase and Excedrin Migraine and sumatriptan on one day each during the intervention. During the baseline phase, P3 reported loud traffic noises (train, ambulances) on one day. P3 noted on one night during the intervention they were disrupted and found they had kicked the blanket off which was related to them feeling hot.

P4 did not report taking any additional medications. P4 reported needing to get up to use the bathroom five nights and being bothered by a loud hotel heater fan and uncomfortable pillows for 2 nights during the baseline phase. P4 reported needing to go to the bathroom 11 nights and being distracted “thinking” on three nights during the intervention phase. During the withdrawal phase, P4 indicated needing to go to the bathroom all nights, having a fever with night sweats two nights, being distracted “thinking” on two nights, experiencing shaking from a booster shot one night, and having a head cold one night.

## 5. Discussion

### 5.1. Insomnia Level

The aim of this study was to determine the impact of weighted blanket use for nighttime sleep on adults with insomnia and sensory sensitivity. All participants in this study experienced a decrease in insomnia per ISI which is similar to the findings of Ekholm et al. [39] who assessed weighted blanket use with adults with insomnia. In contrast to Ekholm et al. [39], this study specifically targeted treatment based on identified participant sensory differences, and Ekholm et al. [39] required their study participants to have one of the following diagnoses: generalized anxiety disorder, major depressive disorder, attention deficit hyperactivity disorder, or bipolar disorder.

### 5.2. Sleep Quality and Duration

The results of this study showed that the use of a weighted blanket during nighttime sleep of adults with insomnia compared to the baseline phase, based on mean score comparisons, resulted in an increase in sleep quality for all participants generally confirming the findings of Ackerley et al. [38] and Green et al. [46]. In addition, this study found an increase in sleep duration for most participants which generally confirms the findings of Ackerley et al. [38]. Like Ackerley et al. [38], the researchers in this study also found a decrease in sleep duration during the withdrawal phase compared to the intervention phase for most participants (P1, P3, and P4), though, again, Ackerley et al. [38] did not use sensory differences as inclusion criteria for the adults with insomnia in their study. These increases in duration and quality were further supported by the aggregated Tau-*U* results from this study comparing baseline to intervention which indicated that the intervention had a moderate effect on sleep quantity and quality achieving significance.

One participant in this study, P2, was an exception to the majority result and reported a decrease in sleep duration during the intervention period as compared to the baseline phase though they did report a decrease in insomnia symptoms postintervention. This participant presented with several differences from the other participants which may have impacted her sleep duration, most notably her occupation as a travel nurse working in intensive care units during the COVID-19 pandemic. In addition, her BMI score placed her in the obese range which, when combined with weighted blanket use during intervention, may have created an obstacle to increased nighttime sleep duration.

Tau-*U* individual results were mixed, and visual analysis examining the impact on duration and quality of sleep overall did not support the effectiveness of the weighted blanket intervention although insomnia levels per ISI improved from pre- to postintervention. Tau-*U* individual results and visual analysis specifically assessed quality and quantity results from the CSDM. Because ISI scores consider multiple factors instead of only duration of sleep or quality, it could be considered more representative of the impact of the intervention on insomnia. What is clear is that neither the quality of sleep nor duration of sleep alone could be isolated as the individual factor that caused the improvement in all participants self-reported insomnia levels from pre- to postintervention. These results illuminate some of the challenges of a sleep study in the natural context where, for example, someone might need to stay up late to study for exams or have a work schedule change that requires an early morning wake-up or attend a social event that runs later than one's typical routine. These are examples of factors that make finding a trendline of increasing duration or a measure of sleep duration alone potentially unrepresentative of an individual's ability to sleep for a certain duration if their life demands allowed it.

### 5.3. Anxiety

In this study, all four participants indicated on the Tuck and Snooze Survey that they frequently worried and had difficulty controlling their worries or sometimes frequently worried and had difficulty controlling their worries as well as indicating they thought anxiety may be contributing to their sleep issues. Three of the four participants (P1, P2, and P3) in this study self-identified as having anxiety and two participants (P1, P2) were diagnosed with or treated for an anxiety disorder in the last year, all of which seems to echo the findings of Carpenter el al. [57] who revealed that sensory over-responsivity in preschool children put them at increased risk for anxiety and associated sleep problems. Though the focus of this study was on sensory sensitivity as a necessary factor for inclusion, the fact that most participants self-identified as having anxiety appears to corroborate the findings of Ekholm et al. [39], who found that weighted blanket use decreased adult insomnia symptom postintervention for those with one of four psychiatric diagnoses, one of which was generalized anxiety disorder. This may suggest that occupational therapists working to evaluate and address the sensory needs of clients and considering providing sensory interventions based on DPT need to consider also screening individuals for anxiety since it appears that a combination of both anxiety and sensory sensitivity may make for the ideal candidate to benefit from these interventions. Certainly, this study adds to the evidence of the interwoven nature of insomnia, anxiety, and sensory differences, particularly sensory sensitivity.

One outlier in this study was P4, who was the only participant who did not self-identify as having anxiety which may explain why P4, though having a decrease in ISI score postintervention resulting in a reduced category of insomnia severity, had the smallest ISI decrease when compared to the other participants. Other factors must also be considered that could explain their smaller decrease on the ISI including that P4 was the oldest participant in this study, potentially perimenopausal, and they also reported frequent nighttime bathroom trips.

### 5.4. Sensory

Prior researchers have suggested that weighted blankets are useful because of the sensory feedback they provide to address specific sensory systems [28–31]. There appears to be a disconnect to this proposed hypothesis and intervention though, since studies of adult use of weighted blankets have not attempted to assess participant sensory differences [38, 39, 46] and evaluate if the blankets are effective for a particular sensory profile. This is concerning since sensory assessment is considered essential to providing appropriate sensory interventions [47].

One of the key findings of this study is that the combination of sensory sensitivity per the AASP with an insomnia level of moderate or severe appears to indicate an adult who may benefit from weighted blanket use. Also, this particular sensory profile combined with self-reported anxiety seems to further illuminate which adult clients could most benefit from weighted blanket use to increase sleep duration and quality and decrease level of insomnia per the ISI. It is also worth noting that these impacts were found utilizing a weighted blanket that was between 6 and 9.6% of participants' body weight.

### 5.5. Limitations

The limitations of this study included a small convenience sample of participants who shared similar demographic characteristics, such as all identifying as female and all but one participant being in their twenties decreasing the generalizability of the results. Results were also dependent on participant accuracy of reporting data on outcome measures. Also, there was baseline phase variability among participants though this may be a factor difficult to eradicate since variability in sleep may well be a component of an individual's sleeping problems. Finally, two of the outcome measures used in this study, the TSS and the ASDQ, were created by the researchers for this study and had no established psychometric properties.

### 5.6. Future Research

Moving forward, a larger study of adults with insomnia with inclusion criteria of anxiety as well as sensory differences much more than the average population in one or more categories on the AASP or similar tool and determining correlations between specific sensory profiles and the impact of the weighted blanket intervention could be helpful in further developing treatment recommendation guidelines. If possible, establishing a stable baseline for each participant would strengthen any results. In addition, lengthening the duration of the intervention may offset any initial adjustment variations that may be expected when altering someone's sleeping habits. Finally, specific investigation of ideal blanket weight in relationship to body weight percentage would further enhance recommendation guidelines for the utilization of weighted blankets to support nighttime sleep.

## 6. Conclusion

This single-case, multiple-participant study was the first to investigate weighted blanket use in adults with moderate to severe insomnia and much more sensory sensitivity than the average population. It is recommended that, for effective sensory intervention to occur, it is crucial that occupational therapists make weighted blanket recommendations based on assessment and matching of interventions to the unique sensory profile of individual clients. The findings of this study suggest that a weighted blanket may be beneficial in reducing insomnia severity and increasing sleep quality and duration for adults who have sensory sensitivity much more than the average population and moderate to severe insomnia, with those who self-report anxiety perhaps experiencing increased benefit.

## Figures and Tables

**Table 1:** Tuck and Snooze Survey.

Gender
Age
Approximate height and weight
Occupation
Do you exercise?
What might this exercise include?
Do you sleep alone or with another person?
Mattress size?
Do you sleep w/ any lights in your bedroom?
Is your sleeping environment completely dark?
Devices that produce sound in your bedroom?
Environmental sounds keeping you up?
Do you worry frequently?
Difficulty controlling worries?
Anxiety contributing to sleep issues?
Currently self-identify as having anxiety?
Have you been diagnosed or treated for anxiety in the past 12 months?
Do you take any prescription medication for anxiety?

**Table 2:** Additional Sleep Diary Questions.

Besides the medications you identified on the Consensus Sleep Diary-Morning questionnaire, did you happen to take any other medicine (prescribed or over the counter) which has side effects that might impact sleep?
If you answered yes to the question above, please identify the medication and dosage.
To your knowledge, were you using the weighted blanket throughout most of the night?
Please note anything that occurred during the evening that disrupted your sleep.
Did you do any exercise yesterday that was outside of what you would consider your normal physical routine?
If you answered yes to the prior question, please share what was different.

**Table 3:** Quantity of sleep mean phase comparisons.

Participant	BL hrs. slept *M* (range)	Tx hrs. slept *M* (range)	*M* diff BL to Tx	W hrs. slept *M* (range)	*M* diff Tx to W
P1	6.07 (4.00-8.33)	7.13 (4.42-9.25)	1.06	6.64 (3.08-9.75)	-0.49
P2	7.52 (6.45-10.00)	6.55 (3.00-10.00)	-0.97	6.85 (5.00-9.00)	0.30
P3	6.62 (5.67-8.02)	7.03 (5.00-8.00)	0.41	6.76 (5.77-8.00)	-0.27
P4	5.29 (4.75-6.00)	6.12 (5.00-7.47)	0.83	5.96 (3.58-7.92)	-0.16

Note. BL = baseline phase; Tx = treatment/intervention phase; *M* = mean; diff = difference; W = withdrawal phase.

**Table 4:** Quality of sleep mean rating phase comparisons.

Participant	BL *M* (category)	Tx *M* (category)	*M* diff BL to Tx	W *M* (category)	*M* diff Tx to W
P1	3.57 (fair)	4 (good)	0.43	4.14 (good)	0.14
P2	2.43 (poor)	3.69 (fair)	1.26	3.57 (fair)	-0.12
P3	2.71 (poor)	2.92 (poor)	0.21	2.29 (poor)	-0.63
P4	2.14 (poor)	2.57 (poor)	0.43	2.29 (poor)	-0.28

Note. BL = baseline phase; Tx = treatment/intervention phase; *M* = mean; diff = difference; W = withdrawal phase. Per CDSM Likert scale rankings 1 = very poor, 2 = poor, 3 = fair, 4 = good, and 5 = very good.

**Table 5:** Individual and aggregated Tau-*U* results for sleep quantity.

Participant or aggregated	Tau	*z*	*p*	90% CI	Effectiveness descriptor
P1	0.31	1.12	0.26	[-0.14<, >0.76]	Moderate
P2	0.19	-0.67	0.50	[-0.64<, >0.27]	None
P3	0.32	1.16	0.25	[-0.13<, >0.77]	Moderate
P4	0.67	2.46	0.01^∗^	[0.22<, >1.00]	Large
Aggregated	0.28	2.03	0.04^∗^	[0.05<, >0.50]	Moderate

^∗^ *p* ≤ 0.05.

**Table 6:** Individual and aggregated Tau-*U* results for sleep quality.

Participant or aggregated	Tau	*z*	*p*	90% CI	Effectiveness descriptor
P1	0.03	0.11	0.91	[-0.42<, >0.48]	None
P2	0.67	2.42	0.02^∗^	[0.21<, >1.00]	Large
P3	0.16	0.60	0.55	[-0.28<, >0.61]	None
P4	0.38	1.38	0.17	[-0.07<, >0.83]	Moderate
Aggregated	0.31	2.25	0.02^∗^	[0.08<, >0.54]	Moderate

^∗^ *p* ≤ 0.05.

## Data Availability

Data supporting the results of this study is not available to protect participant privacy.
